# Multimodality imaging in lobular breast cancer: Differences in mammography, ultrasound, and MRI in the assessment of local tumor extent and correlation with molecular characteristics

**DOI:** 10.3389/fonc.2022.855519

**Published:** 2022-08-22

**Authors:** Bartosz Dołęga-Kozierowski, Michał Lis, Hanna Marszalska-Jacak, Mateusz Koziej, Marcin Celer, Małgorzata Bandyk, Piotr Kasprzak, Bartłomiej Szynglarewicz, Rafał Matkowski

**Affiliations:** ^1^ Breast Unit, Department of Breast Imaging, Lower Silesian Oncology, Pulmonology and Hematology Center, Wroclaw, Poland; ^2^ Burn and Plastic Surgery Department, Ludwik Rydygier Memorial Specialized Hospital in Krakow, Krakow, Poland; ^3^ Department of Anatomy, Jagiellonian University Medical College, Krakow, Poland; ^4^ Breast Unit, Department of Breast Surgery, Lower Silesian Oncology, Pulmonology and Hematology Center, Wroclaw, Poland; ^5^ Department of Oncology, Faculty of Medicine, Wroclaw Medical University, Wroclaw, Poland

**Keywords:** breast cancer, invasive lobular carcinoma (ILC), invasive lobular breast cancer, magnetic resonance imaging, multimodality imaging, lobular breast cancer

## Abstract

**Introduction:**

Invasive lobular breast cancer (ILC) is a diagnostic challenge due to the diversity of morphological features. The objective of the study was to investigate the presentation and local extent of ILC using various imaging techniques and to assess the correlation between imaging and molecular profile.

**Materials and methods:**

We reviewed 162 consecutive patients with ILC found on vacuum-assisted biopsy, who underwent evaluation of the lesion morphology and extent using ultrasound (US), mammography (MMG), and magnetic resonance imaging (MRI). Radiographic features were compared with ILC intrinsic subtype based on the expression of Ki-67 and estrogen, progesterone, and HER2 receptors.

**Results:**

A total of 113 mass lesions and 49 non-mass enhancements (NMEs) were found in MRI. Masses were typically irregular and spiculated, showing heterogeneous contrast enhancement, diffusion restriction, and type III enhancement curve. NMEs presented mainly as the area of focal or multiregional distribution with heterogeneous or clumped contrast enhancement, diffusion restriction, and type III enhancement curve. Lesion extent significantly varied between MRI and MMG/ultrasonography (USG) (P < 0.001) but did not differ between MGF and ultrasonography (USG). The larger the ILC, the higher the disproportion when lesion extent in MRI was compared with MMG (P < 0.001) and ultrasonography (USG) (P < 0.001). In the study group, there were 97 cases of luminal A subtype (59.9%), 54 cases of luminal B HER2− (33.3%), nine cases of luminal B HER2+ (5.5%), and two cases of triple negative (1.2%). The HER2 type was not found in the study group. We did not observe any significant correlation between molecular profile and imaging.

**Conclusion:**

MRI is the most effective technique for the assessment of ILC local extent, which is important for optimal treatment planning. Further studies are needed to investigate if the intrinsic subtype of ILC can be predicted by imaging features on MRI.

## Background

Breast cancer imaging is constantly evolving and research protocols are continually being modified. The method of highest sensitivity (94% to 99%) for invasive lobular breast cancer (ILC) detection is magnetic resonance imaging (MRI) (1–3). Although ILC accounts for 5% to 15% of all breast cancers (4) due to its course and wide diversity of histopathological, clinical, and radiological images, ILC still presents significant challenge for clinicians specializing in breast oncology (2, 5, 6).

Biological diversity of ILC is reflected in molecular subtypes defined on the basis of standard biomarkers analyzed *via* immunohistochemistry: estrogen receptor (ER), progesterone receptor (PR), and human epidermal growth factor-2 receptor (HER2) as well as the estimation of tumor proliferation index Ki67 that allows risk stratification and implementation of personalized therapies. The modern classification of lobular breast cancer (LBC) includes five subtypes of different molecular profile: luminal A (ER+ and/or PR+, HER2−, Ki67<15%), luminal B (HER2− subtype: ER+ and/or PR+, HER2−, Ki67≥15%; HER2+ subtype: ER+ and/or PR+, HER2+), HER2 type (ER− and PR−, HER2+), and triple negative (TN) breast cancer (7).

Because of their radiological characteristics, lesions that cannot be seen in mammography (MMG) or in ultrasound (US) are revealed to be advanced when they are exhibited in MRI. Moreover, ILC is often multifocal, multicentric, or even bilateral, each of which influences choice of therapeutic procedure. That is why, for many years, MRI has been recognized as a diagnostic standard for ILC as it has the highest sensitivity among the available imaging methods (2, 8–10).

Magnetic resonance imagining is often emphasized as the LBC diagnosis of choice because it can easily detect changes that other methods often cannot (11). One factor influencing MRI’s popularity is that lobular carcinoma spreads along milk ducts and the loss of E-cadherins in terminal duct lobular units. This type of growth is characterized by much lower incidence of necrotic changes, hemorrhages, or microcalcifications when compared to ductal carcinoma *in situ* (2, 5, 12, 13).

The correlation between radiological features and molecular profile of the LBC is the subject of extensive research, the results of which do not allow clear conclusions to be drawn due to relatively small study groups (11, 14–17).

## Study objectives

1. The assessment of morphology and local extent of ILC in three imaging techniques.
2. The assessment of the correlation between the results of three imaging methods (MMG, US, and MRI) and molecular profile of ILC.

## Materials and methods

One hundred sixty-two patients with ILC diagnosis who were treated in the Breast Unit of Wrocław Comprehensive Cancer Centre, Wroclaw, Poland, between September 2016 and February 2020 were subjected to a retrospective analysis of their imaging (MRI, US, and MMG) and histological test results.

The diagnosis of ILC was made according to the following protocol: patients were referred to the Breast Unit of Wrocław Comprehensive Cancer Centre to check a lesion discovered during outpatient US examination. Shortly thereafter, US and MMG were performed in the Wroclaw breast unit. Results were analyzed by two independent teams of specialists using American College of Radiology Breast Imaging Reporting and Data System, and patients were qualified for percutaneous core needle biopsy. Ultimately, the study included only patients with ILC confirmed in the histopathological examination.

The criteria for exclusion of patients from the study included neoadjuvant chemotherapy, allergy to gadolinium, and other medical contraindications to contrast-enhanced MRI. US and MMG scans were analyzed by three breast radiologists with at least 20 years of professional experience and one assistant with 4 years of professional experience, whereas the breast MRI scans were subjected to independent dual review by radiologists interpreting more than 600 breast MRI scans per year.

### Ultrasound

Esaote My Lab Class C ultrasound devices and a 5- to 13-MHz linear probe were used to perform US examinations. The default “breast” preset was used for the analysis of images, which guaranteed repeatability of the tests. In addition, single focusing was used. The test result was prepared according to ACR BI-RADS.

### Mammography

Mammographic examination was performed on the Hologic Selenia Dimension system (Hologic, Inc., Bedford, MA) in standard projections in the compression force range of 90N to 140N and then described on the Hologic console with Secure View software according to ACR BI-RADS lexicon. The measurement was performed as follows: round or oval tumor (main lesion – index mass), spiculated lesion (main lesion without projections), and area of high density (borders of the highest saturation area).

The description included isolated alterations of cell architecture and accompanying changes, as well as microcalcifications measured for greatest extent.

### Magnetic resonance imaging

MRI of the breast was performed on the Magnetom Avanto Tim Dot 1.5T (Siemens Healthcare, Erlangen, Germany) with a compatible 18-channel diagnostic breast coil. Imaging was performed within 14 days of core needle biopsy and with the patient in the prone position. The tests were conducted according to the following protocol:

T1 HR—slice thickness, 0.7 mm [voxel size: 0.7 × 0.7 × 0.7 mm; SNR (signal-noise ratio), 1.00]; slices per slab, 208; TR, 5.64 ms/TE, 5.64 ms; FoV read, 250 mm; FoV phase, 169.3; bandwidth = 300 Hz; slice gap, 0.14 mm; flip angle, 15**°**; and a total acquisition time = 2 min 28 s.

T2—slice thickness, 2 mm (voxel size: 0.5 × 0.5 × 2.0 mm; SNR, 1.00); slices, 57; TR, 6,670 ms/TE, 100 ms; FoV read, 250 mm; FoV phase, 168.8; bandwidth = 326 Hz; slice gap, 0.4 mm; flip angle, 150**°**; and a total acquisition time = 2 min 53 s.

TIRM (Turbo Invertion Recovery Magnitude)—slice thickness, 2 mm (voxel size: 0.7 × 0.7 × 2.0 mm; SNR, 1.00); slices, 57; TR, 7,850 ms/TE, 63 ms; FoV read, 250 mm; FoV phase, 168.8; bandwidth = 334 Hz; slice gap, 0.4 mm; flip angle, 150**°**; and a total acquisition time = 3 min 24 s.

T1 3D dynamic—matrix, 389 × 256; slice thickness, 1 mm (voxel size: 1 × 1 × 1 mm; SNR, 1.00); slices per slab, 144; TR, 4.42 ms/TE, 1.7 ms; flip angle, 10°; acquisition time of each phase, approximately 55 s (one phase before contrast, six phases after contrast injection).

DWI—b-value, 50/400/800 s/mm^2^; slice thickness, 3 mm (voxel size: 1.3 × 1.3 × 3.0 mm; SNR, 1.00); slice numbers, n = 45; TR, 6300 ms/TE, 70 ms; slice gap, 0.6 mm; and a total acquisition time = 3 min 22 s.

The dynamic test was performed with the administration of the contrast agent Dotarem (gadoterate meglumine) at the dose of 0.1 mmol/kg and the flow of 2 ml/s, followed by a rinse with 30 ml of NaCl.

The tests were analyzed by independent radiology specialists (double reading) using Siemens software tool (Brevis MRI), and all lesions were evaluated by the American College of Radiology – BIRADS breast MRI lexicon (Fifth Edition).

For all axial plane acquisitions, the phase encoding direction was from right to left to limit artifacts repeating cardiac and respiratory movement. Moreover, movement artifacts were eliminated by the “Motion Correction” function. “Color mapping” function allowed confirmation of the locations for determining the enhancement curves.

In first step, we placed Region of interest (ROI) on the aorta to confirm a typical washout pattern. Subsequently, the enhancement curve was assessed in the initial phase and next in the late phase. In the T1 3D dynamic, the first two phases are the sequences that were used to assess the morphology of the lesion and to determine the inflow of contrast in the initial phase (where we defined the inflow as slow <50%, medium 50%–100%, and fast > 100%). The remaining acquisitions were used to determine the type of washout curve: type 1, benign; type 2, intermediate with plateau; type 3, malignant, with secondary washout.

In the dynamic sequence, ROI (size, 3 × 3 pixels) was measured three times on hyperintense lesions in DCE-MRI, both within mass and non-mass enhancement (NME). Lesion size was measured on the DCE MRI images. An apparent diffusion coefficient (ADC) was calculated from DWI by using a monoexponential model in dedicated and clinically validated software syngo.MR BreVis (Siemens Helthineers Erlangen Germany) using standardized DWI preprocessing pipeline that included all necessary steps in particular epi-distortion and motion correction.

To determine the ADC value, we looked for pathological contrast enhancement—tumor mass or NME, which correlated with the hyperintensive region in the DWI (b = 800 s/mm^2^) and the low signal on the ADC maps. Afterward, ROI about 5 ± 2 mm^2^ was placed two times on the most restricted area inside the solid part of the lesion on the ADC map. We were trying to avoid cystic, necrotic, fatty regions, or hematoma after biopsy inside the mass using T2-weighted images or TIRM.

## Statistical analysis

Quantitative data were reported as mean ± standard deviation (SD) or median/interquartile range, according to a normal distribution. For the qualitative data, frequencies and percentages were calculated. Mean differences between the two groups were compared by the Student’s t-test, whereas the Mann–Whitney U-test was applied for comparisons of median values. The qualitative variables were compared using the chi-squared (χ2) test of proportions for categorical variables. The receiver operating characteristic (ROC) curves were used to assess both MRI and MMG method to better discriminate nodal status (presence of metastases based on US, widely regarded as the gold standard for tumor detection). Area under the curve (AUC) with standard error (SE) was reported, as well as sensitivity and specificity. In addition, the Spearman correlation between parameters was performed. Bland–Altman method was used to compare two radiological methods in measuring lesion size. The data were analyzed using StatSoft Statistica 13.1 PL for Microsoft Windows 10. The results with P < 0.05 were considered as statistically significant. Correspondence analysis was performed to compare the relative frequencies of prevalence of selected features across different imaging methods.

## Results

### Mammography

In a study group of 162 patients, 54 cases of tumor and 20 cases of high density areas were found (**Table 1**). The sensitivity of MMG in the diagnosis of ILC in the study group was 113 of the 162 (69.8%). Areas of high density identified by MMG most often did not correspond to morphological changes of the NME identified by MRI. Moreover, the size of the lesions found in the two methods was different (P = 0.007).

**Table 1:** Imaging parameters and clinicopathological features of 162 patients with invasive lobular carcinoma.

Demographic data												
Patients count			162									
Patients age (min/max/average)			32/94/65.5									
MRI: mass			MRI: non-mass enhancement (NME)						MRI: other			
**Shape**		**113**	**Distribution**				**49**		**Architectural distortion**			**162**
Oval		8	Focal				18		None			159
Round		4	Linear				4		Present			3
Irregular		101	Segmental				6		**Lymph nodes**			**162**
**Margin**		**113**	Regional				4		Normal			146
Circumscribed		8	Multiple regions				17		Abnormal			16
Not circumscribed		105	Diffuse				0					
**Enhancement**		**113**	**Enhancement**				**49**					
Homogeneous		28	Homogeneous				8					
Heterogeneous		81	Heterogeneous				22					
Rim enhancement		4	Clumped				15					
Dark internal septations		0	Clustered ring				4					
**Kinetic curve (delayed phase)**		**113**	**Kinetic curve (delayed phase)**				**49**					
Persistent		18	Persistent				8					
Plateau		34	Plateau				8					
Washout		61	Washout				33					
**Mammography (MMG)Ultrasound (US)**												
**Lymph nodes**			**162**				**Lymph nodes**					**162**
Normal			154				Normal					142
Abnormal			8				Abnormal					20
**Findings**			**74**				**Findings**					**161**
Mass			54				Mass					144
Asymmetric density			20				Region					17
**Calcifications**			**162**				**Margin**					**155**
None			128				Circumscribed					3
Present			34				Not circumscribed					152
**Architectural distortion**			**162**									
None			129									
Present			33									
** Histopathology and immunohistochemistry**												
**Molecular subtypes**			**162**				**Grading (G)**					**162**
Luminal A			97				G1					12
Luminal B (HER2−)			54				G2					143
Luminal B (HER2+)			9				G3					7
HER2 type			0									
Triple negative			2									
**Comparison of results between three imaging modalities**												
			**Total cases**		**Average**		**Median**	**Min**			**Max**	**Std Dev**
**Mass** (mm)	MRI		**113**		35		30	5			122	24
	US				24		22	3			65	13
	MMG				24		20	1			84	15
**NME** (mm)	MRI		**49**		59		60.5	15			96	24
	US				31		26	0.9			84	18
	MMG				31		20.5	8			84	23
**Receptors and markers statistics**												
**Tested positive**			**Total cases**	**Average**		**Median**	**Min**	**Max**		**Low**	**Up**	**Std Dev**
Estrogen receptor ER (%)			162	93.97		100	0	100		90	100	15.14
Progesterone receptor PR (%)			162	58.70		75	0	100		8	100	41.07
Ki-67 biomarker (%)			162	13.37		10	0	70		5	20	11.11

### Ultrasound

In US, a mass was found in 144 of the 162 patients (mean size, 22.6 mm; range, 9–84 mm), and an area of indefinite shape and borders were detected in 17 patients (mean size, 28.6 mm; range, 10–87 mm). The sensitivity of US in the diagnosis of ILC in the study group was 161 of 162 (99.4%). The vast majority of changes (93.8%) revealed poorly defined contours. It was also discovered that areas of concern revealed by US do not correspond accordingly to NME morphological changes isolated by MRI.

### Magnetic resonance imaging

**Figure 1:**
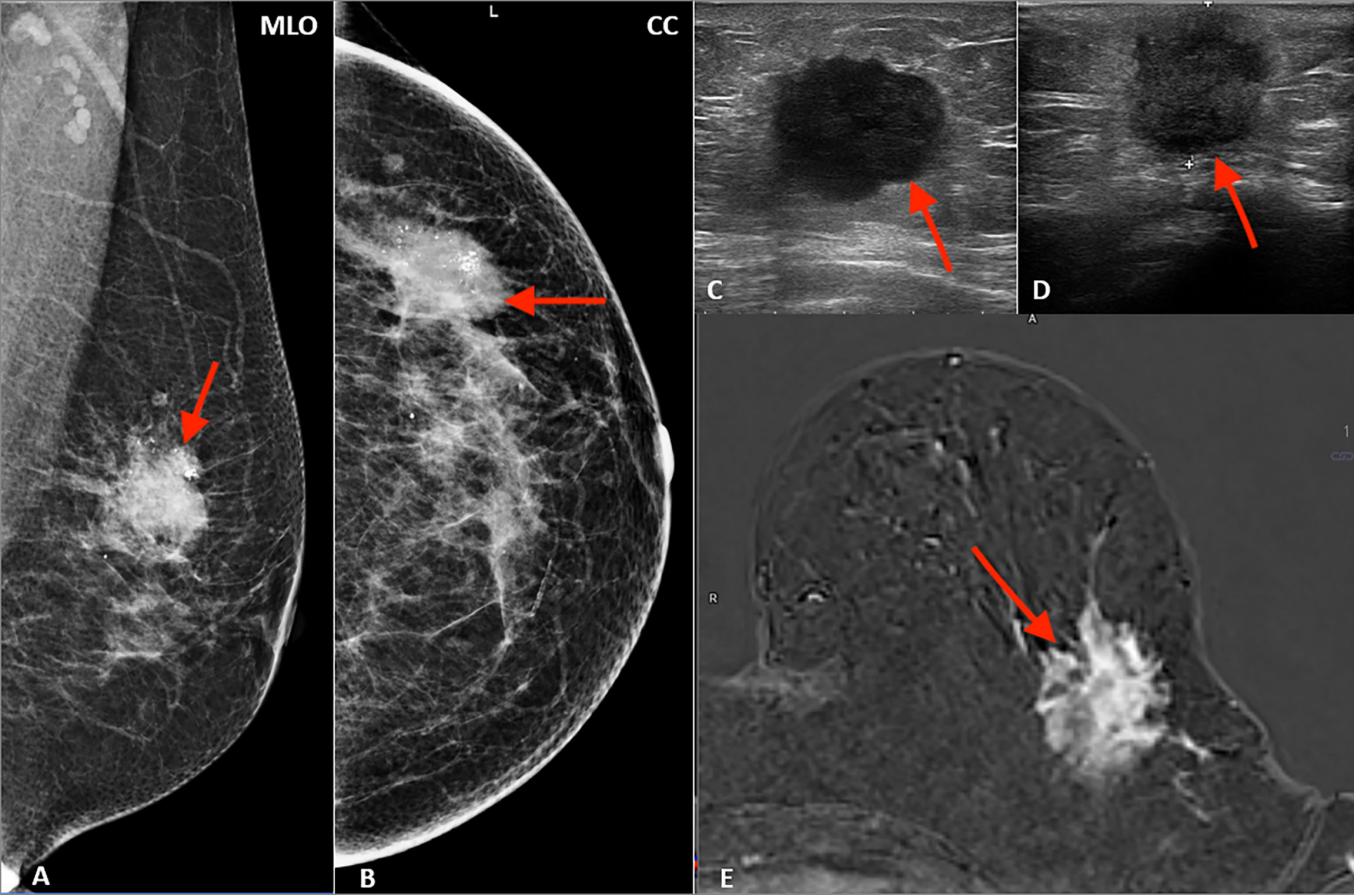
Multimodality presentation of lobular breast cancer; Patient 1 left breast: **(A, B)** mammography: MLO **(A)** and CC **(B)**—not circumscribed, spiculated mass with microcalcifications (red arrow); **(C, D)** ultrasound—not circumscribed, spiculated, hypoechoic mass (red arrow); **(E)** MRI T1 post contrast—not circumscribed, spiculated mass with heterogenous contrast enhancement (red arrow).

**Figure 2:**
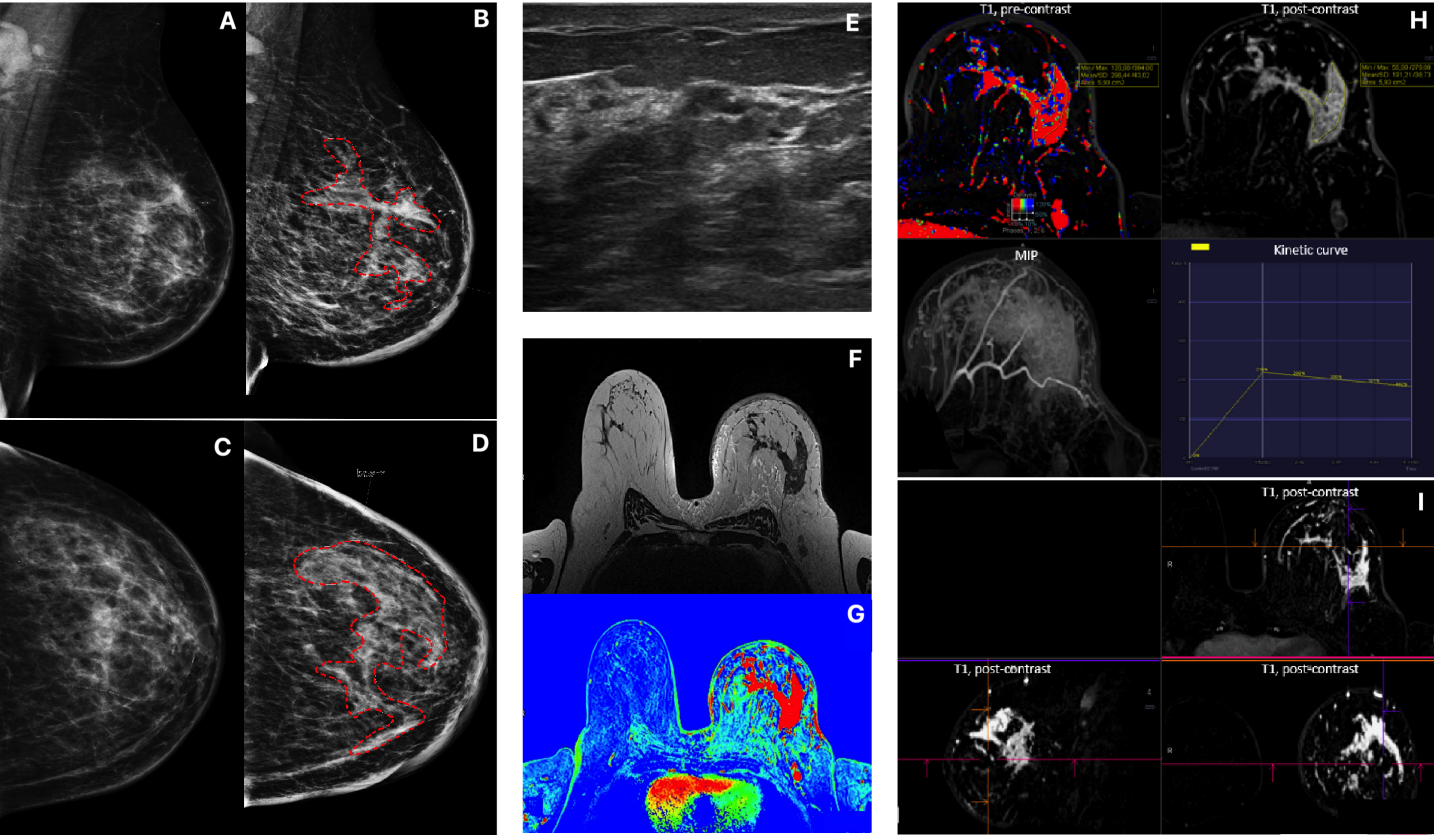
Patient 2, Multimodality presentation of Lobular Breast Cancer, left breast: **(A, B)** Mammography, [**(A)** MLO and **(B)** CC] asymetric density cancer, cancer marked with red dotted line. **(C, D)** Mammography, [**(C)** MLO and **(D)** CC] the same Patient after 4 months, cancer marked with red dotted line. **(E)** ultrasound- not circumscribed, hypoechoic region. **(F)** MRI T2 TSE - non-mass enhancement. **(G)** MRI T1 fl3d dynamic PEI. **(H, I)** MRI - diffused, non-mass enhancement with heterogenous enhancement [**(H)** T1 pre contrast; T1 post contrast; MIP and wash out kinetic curve; **(I)** 3D T1 post contrast].

In a study group of 162 patients with ILC, there were 113 tumors (69.8%) and 49 (30.2%) NME-type changes found in MRI. The most common morphological type of ILC was an irregular, spiculated mass showing heterogeneous contrast enhancement, diffusion restriction of the mean ADC value of 0.74 × 10–3 mm^2^/s, and type III enhancement curve. In NME changes, the types of distribution most often found were focal (36.7%) and multi-regional (34.7%), with heterogeneous and clumped contrast enhancement accounting for 44.9% and 30.6%, respectively, of all NME changes. In NME changes, the dominant type of enhancement was type III with the mean ADC value of 0.72 × 10^3^ mm^2^/s (**Table 1**; **Figures 1**, **2**).

### Histopathology

**Figure 3:**
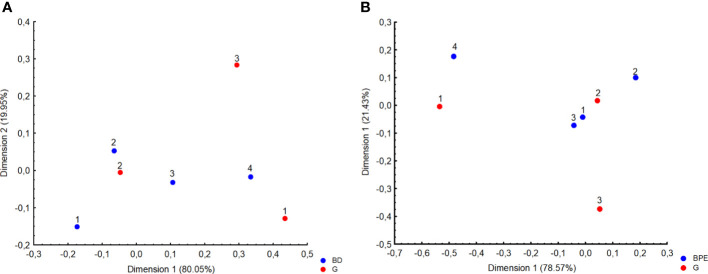
Correspondence analysis plot of the data which is a two-dimensional representation of grading (G) and **(A)** Breast Density (BD 1, almost entirely fat; 2, scattered fibrograndular tissue; 3, heterogenous fibrograndular tissue; 4, extreme fibrograndular tissue), as well as **(B)** background parenchymal enhancement (BPE).

The dominant type of tumor identified in histopathology was of grade (G feature) 2 (88.3%) with no amplification of HER2 receptors (92%). The mean value of receptor expression in the study group was, respectively, ER – 93.9% and PR – 58.7%, whereas the mean Ki67 value was 13.37%, which is related to the higher incidence of luminal A subtype in the study group (n = 97, 59.9%). The data on the mass feature G, structure, and background parenchymal enhancement (BPE) show that G2 tumors with heterogeneous fibroglandular structure and slight enhancement in the stroma are more common (**Table 2**; **Figure 3**).

**Table 2:** Comparison between feature G *vs*. MRI and feature G *vs*. BPE.

Breast density	G1	G2	G3	Total
Almost entirely fat	2	19	0	21
Scattered fibrograndular tissue	4	77	4	85
Heterogenous fibrograndular tissue	4	36	2	42
Extreme fibrograndular tissue	2	11	1	14
**Total**	**12**	**143**	**7**	**162**
**BPE**	**G1**	**G2**	**G3**	**Total**
1. Minimal	6	68	4	78
2. Mild	1	37	1	39
3. Moderate	3	30	2	35
4. Marked	2	8	0	10
**Total**	**12**	**143**	**7**	**162**

### Imaging and assessment of local extent

It is interesting that, in the study group, microcalcifications were found in 34 of 162 (21%) patients in MMG. In the group of changes presenting as tumors in MRI, microcalcifications were found in 20 of 113 (17.7%) patients in MMG, whereas in the NME group, they were significantly more often, i.e., found in 14 of 49 (28.6%) patients (P < 0.05).

The size of the lesions described in MRI did not differ significantly from those described by US examination (P = 0.056).

**Figure 4:**
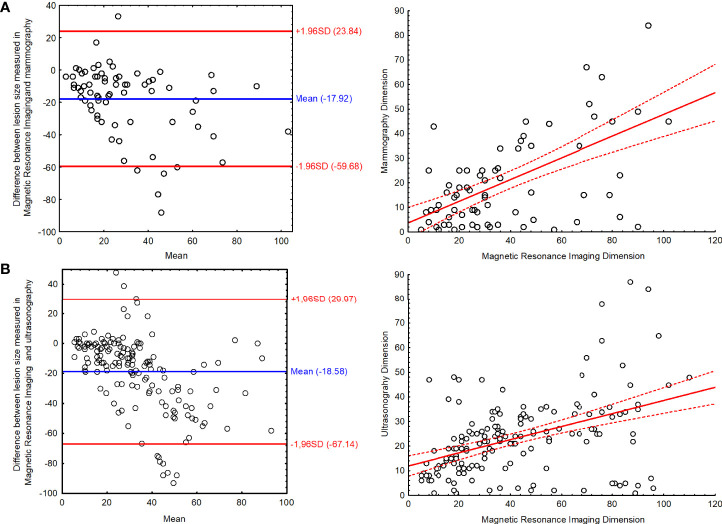
The Bland–Altman plot with scatter plot for the results of lesion size measured in magnetic resonance imaging and **(A)** mammography and **(B)** ultrasonography.

It has also been observed that the bigger the lesion, the higher the disproportion between its size measured in different methods: MRI *vs*. MMG R = 0.455; P < 0.001 and MRI *vs*. US R = 0.425; P < 0.001). The Bland–Altman plot along with scatterplot graph is presented in **Figure 4**. The limits of agreement for the lesion measured in MRI and MMG varied from −59.68 to 23.84 mm and for MRI and US varied from −67.14 to 29.97 mm (**Figure 4**)

In the studied patients with T1 tumor found *via* US (n = 57), MRI showed that the size of the lesion was underestimated and the T feature of the lesion increased to T2 in 23 cases (41%) and to T3 in two cases (3.6%). A similar situation occurred in patients who had T2 tumor found in US (n = 92), and the lesion was reassessed as T3 in 35 cases (38%).

In 50 (30.8%) cases, MMG did not reveal any pathological changes (tumors, high-density areas, microcalcifications, and/or architectural alterations).

In the analyzed material, NME lesions were characterized by a higher range of sizes and were not as homogeneous as tumors.

Comparison of the ADC value and the tumor’s T feature according to the TNM classification showed statistically significant difference between the T1 and T3 group of tumors. The higher the tumor’s T feature, the lower the ADC value, which corresponds to increased diffusion restriction.

Architectural alterations were found in 20.4% of patients in MMG. Molecular studies revealed association on the verge of statistical significance between architectural alterations and increased expression of progesterone receptors (P = 0.57). Other results of molecular studies do not correlate with architectural alterations (**Table 4**). Architectural alterations found in MMG were confirmed in MRI in two patients only.

### Imaging and molecular profile

Apart from the results presented here, no findings proved correlation between ILC presentation on imaging and molecular profile of the tumor. Microcalcifications did not correlate with the expression of HER2 receptors (P = 0.87), ER (P = 0.81), PR (P = 0.65), or Ki67 (P = 0.25). The same is true for architectural alterations that do not correlate with the expression of HER2 (P = 0.4), ER (P = 0.4), or Ki67 (P = 0.85). Similarly, morphological type of ILC revealed in MRI did not correlate with ADC value (P = 0.62) (**Table 3**).

**Table 3:** Comparison between presence of microcalcifications and molecular tumor profile.

Variable		No Microcalcifications (n = 34)		Present Microcalcifications (n = 128)		
	Mean (SD)	Range	Mean (SD)	Range	p
**ER**	92.9	10–100	94.2	0–100	0.82
**PR**	55.1	0–100	59.6	10–100	0.66
**KI67**	14.6	1–60	13	5–19	0.26

### Differences between luminal A and luminal B

In the study group consisting of 162 patients, there were 97 cases of luminal A subtype (59.9%), 54 cases of luminal B HER2− (33.3%), nine cases of luminal B HER2+ (5.5%), and two cases of triple negative (1.2%). The HER2 type was not found in the study group (**Table 4**).

**Table 4:** Differences between luminal A and luminal B ILC type.

**Demographic data**																
						**Luminal A (Lum A)**							**Luminal B (Lum B)**			
**Patients count**						97							63			
**Patients age (min/max/average)**						46/91/66							32/94/64.5			
**General data**																
**Size distribution (feature T) in various methods**			**T1size < 2 (cm)**			**T22 (cm) ≤ size < 5 (cm)**		**T3size ≥ 5 (cm)**		**Total cases**			**Grading (feature G)**		**Lum A**	**Lum B**
**Lum A**		MRI	22			45		30		97			G1		6	6
		MMG	78			16		3					G2		89	52
		US	45			46		6					G3		2	5
**Lum B**		MRI	14			28		21		63						
		MMG	40			19		4								
		US	25			35		3					Total cases		97	63
**MRI: mass**						**MRI: non-mass enhancement (NME)**							**MRI: other**			
			**Lum A**		**Lum B**			**Lum A**			**Lum B**				**Lum A**	**Lum B**
**Shape**						**Distribution**							**Architectural distortion**			
Oval			6		2	Focal		11			7		None		89	55
Round			3		1	Linear		3			1		Present		8	8
Irregular			56		43	Segmental		4			2		**FGT**			
**Margin**						Regional		3			1		Fat		14	6
Circumscribed			6		2	Multiple regions		11			6		Scattered		49	35
Not circumscribed			58		43	Diffuse		0			0		Heterogeneous		26	16
Spicular			1		1	**Enhancement**							Extreme		8	6
**Enhancement**						Homogeneous		4			4		BPE			
Homogeneous			22		5	Heterogeneous		15			7		Minimal		49	28
Heterogeneous			43		41	Clumped		10			5		Mild		21	18
**Kinetic curve (delayed phase)**						Clustered ring		3			1		Moderate		19	15
Persistent			15		3	**Kinetic curve (delayed phase)**							Marked		8	2
Plateau			17		16	Persistent		6			2					
Washout			33		27	Plateau		6			2					
						Washout		18			13					
**Mammography (MMG)**								**Ultrasonography (US)**								
				**Lum A**		**Lum B**							**Lum A**		**Lum B**	
**Calcifications**								**Margin**								
None				78		48		Circumscribed					1		2	
Present				19		15		Not circumscribed					88		57	
**Architectural distortion**								Irregular					4		1	
None				77		50		Spicular					0		0	
Present				20		13		**Lymph nodes**								
**Lymph nodes**								Normal					86		54	
Normal				94		58		Abnormal					11		9	
Abnormal				3		5		**Findings**								
**Findings**								Mass					85		57	
Mass				29		25		Region					11		6	
Asymm. density				11		9										
**Size and diffusion statistics**																
**Characteristic**				**Total cases**			**Average**	**Median**	**Min**			**Max**	**Low**	**Up**	**Std Dev**	
**mass MRI**	Mass size (cm)			Lum A		65	3.41	2.80	0.50			9.80	1.80	4.50	2.31	
				Lum B		46	3.66	3.10	0.60			12.20	1.80	4.40	2.68	
	Mass ADC[10^−3^ mm^2^/s]			Lum A		65	0.73	0.70	0.30			1.20	0.60	0.80	0.17	
				Lum B		46	0.76	0.75	0.40			2.00	0.60	0.90	0.28	
**NME MRI**	NME size (cm)			Lum A		32	5.82	5.55	2.00			11.00	3.60	8.05	2.60	
				Lum B		17	6.24	7.10	1.50			9.00	4.40	8.30	2.35	
	NME ADC[10^−3^ mm^2^/s]			Lum A		32	0.74	0.72	0.00			1.20	0.60	0.87	0.24	
				Lum B		17	0.68	0.70	0.30			1.20	0.50	0.70	0.23	
**MMG**	Mass size (cm)			Lum A		29	1.65	1.10	0.10			8.40	0.50	2.20	1.73	
				Lum B		25	3.03	2.50	0.10			8.40	1.50	4.50	1.98	
	asymmetric density size (cm)			Lum A		11	1.52	0.80	0.10			6.70	0.20	1.60	1.99	
				Lum B		9	1.48	1.10	0.20			4.40	0.30	1.60	1.51	
**US**	mass size (cm)			Lum A		85	2.13	2.10	0.10			8.40	1.10	2.70	1.51	
				Lum B		57	2.46	2.30	0.20			6.40	1.20	3.30	1.53	
	region size (cm)			Lum A		11	2.85	2.80	0.10			5.60	2.20	3.70	1.59	
				Lum B		6	2.90	1.95	0.10			8.70	1.30	3.40	3.05	

### Lymph nodes

**Figure 5:**
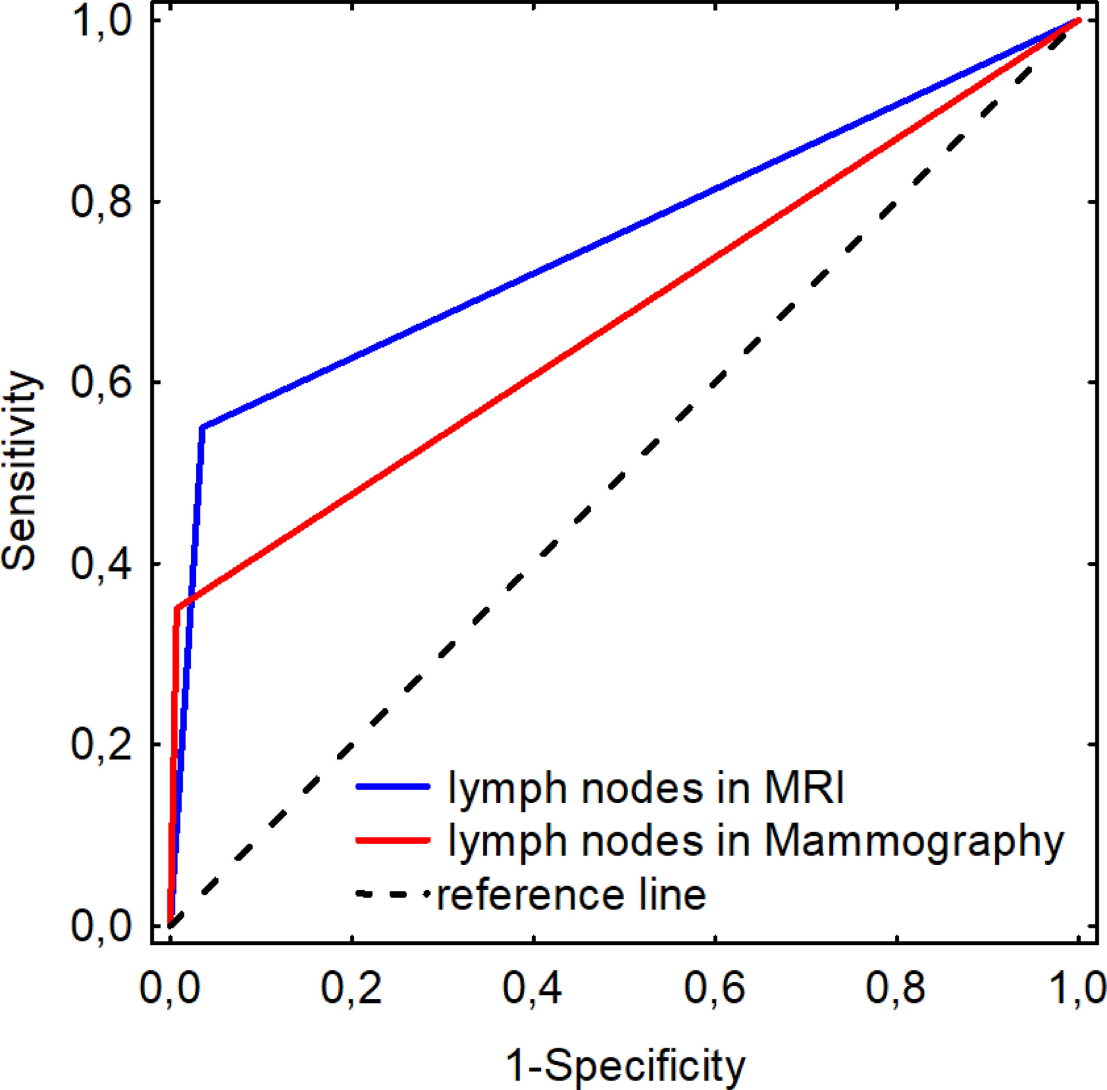
ROC curves for detecting lymph nodes in MRI and mammography.

In the study group, pathological lymph nodes were found in 20 patients (12.3%). US had the highest detection rate for pathological lymph nodes (US 12.3% vs. MRI 9.9% vs. MMG 4.9%) (MRI AUC 0.757 ± 0.071; MMG AUC 0.671 ± 0.076). MRI was shown to be more sensitive than MMG with similar specificity in imaging pathological nodes in patients with ILC when assessed using ROC curve (MRI sensitivity = 0.550; specificity = 0.965; MMG sensitivity = 0.350; specificity = 0.993) (**Figure 5**).

## Discussion

Interestingly, this study confirms the findings of others regarding how MMG and US underestimate LBC size while MRI produces more accurate data. These findings suggest that MRI is the best choice during pre-operative management so as to customize the most appropriate therapeutic plan for the individual patient. (18–20) Some works suggest that ILC is often multifocal or even bilateral, but it is often not detected in methods other than MRI. Schelfout et al. demonstrated in their study that additional ILC foci, previously not shown by US or MMG, were detected in 50% of patients. (21) In addition to MRI’s ability to most clearly define tumor parameters, this study also identified its ability to assess lesion size more accurately than other methods. This finding is important because this information affects the T feature of TNM classification and consequently changes therapeutic management. (22)

In addition, the image of ILC in MRI was the same as described in the literature. According to the authors, ILC presents most often as an irregular, spiculated tumor showing a washout enhancement curve type. However, one should remember that this picture is not pathognomonic for ILC and may correspond to invasive ductal carcinoma (IDC). (4, 18, 23)

One of the diagnostic problems described in the available literature is the determination of ADC value for NME changes. (24) It should be noted, however, that although the T feature of the tumor correlates with the ADC value obtained in MRI, the literature, the same as the presented study, does not show any association between molecular profile of the tumor and ADC value.14 Therefore, ADC value is suitable only to indicate potential malignancy of the tumor, but it cannot be used to predict its molecular profile when standard examination protocol is followed.

An MRI may present LBC in two forms: as a mass or NME. The percentage distribution of these changes in the described database is consistent with the data from the literature: – 5% to 69% for NME and 31% to 95% for the tumor. (25–28) In the study group, LBC most often appeared as a mass in MMG. The second most frequent manifestation of LBC was architectural alteration. The obtained results regarding the morphology of LBC in imaging are consistent with the data from the literature. (13, 29)

Moreover, some data from the literature suggest a high rate of false negative results in MMG, as high as 29% of ILC cases. (30) This finding is probably due to the fact that the only presentation of ILC in MMG may be architectural alterations without the mass. (31) Approximately 50 cases (30.8%) of our study group had tumors that were impossible to identify, as were their associated pathological areas. In the described study, architectural alterations found in MMG were not visible in MRI. One of the reasons for this invisibility is the structure of breast tissue and it confirms that MRI is not a method of choice for the assessment of architectural alterations of the breast. (32)

Another factor that hinders the diagnosis of ILC in MMG is the relatively rare occurrence of microcalcifications. According to the literature, microcalcifications occur in only one to 25% of all ILC cases, which has a negative effect on the sensitivity of MMG in detecting this type of lesion (33–35). This finding is probably due to the fact that ILC does not invade milk ducts and, consequently, does not contribute to the formation of microcalcifications (13). At the same time, it should be noted that in the study group, microcalcifications were found in as many as in 42.85% of G3 tumors. It should be remembered, however, that there were too few patients with G3 tumor to consider these results statistically significant. The available literature indicates the much lower incidence of microcalcifications in ILC compared to IDC, which may be a predictive factor in the assessment of the tumor’s G feature. This indication, however, requires further study on larger groups of patients (29).

In the study group, most LBC cases confirmed in MRI (99.38%) were also revealed in the US. Studies on the usefulness of US indicate its high sensitivity in the detection of LBC, defined by the authors at 68% to 95% (36–39). At the same time, the literature emphasizes the issue of accurate assessment of lesion size, an issue also highlighted in the presented work. The characteristics of LBC, particularly in this histological type of cancer, significantly complicate the assessment of the lesion borders and extent, which is often associated with underestimated size of the tumor (20, 40).

Literature mentions some attempts to find the association between radiological image and receptor profile of the tumor. Dilorenzo et al. reported attempts to demonstrate the relationship between BPE type and clinical tumor subtype. (41) This relationship, however, has not been found in the study group. Moreover, King et al. suggested in their work increased incidence of ILC in patients with high BPE. (42) Ko et al. found higher incidence of NME lesions in patients with the breast cancer (BC) HER2+ type (43).

Wen et al. correlated their findings with US images of the lesions. Interestingly, imaging did not reveal any differences between luminal A and luminal B morphologies, a finding consistent with ours. Wen et al. described the differences in the morphology between HER2-type tumors and the luminal type. Although the study by Wen et al. included a large number of study participants, none were experiencing HER2 tumors; therefore, data could not be compared between the study by Wen et al. and this one (44).

Despite the abovementioned limitations of US in the assessment of ILC size, it remains the gold standard in the diagnosis of changes in the lymph nodes. In the study group, US identified the largest number of pathological lymph nodes and was considered the gold standard in the evaluation of other methods (45, 46). The study is consistent with the literature data and it shows high sensitivity and specificity of MRI in the assessment of lymph nodes.

One limitation encountered during this study is the inability of MRI to correlate lesion size with its actual size as assessed *via* histopathology. This discrepancy was caused by retrospective methodology of the study and the fact that some patients received neoadjuvant chemotherapy that affected the size of the tumor before the surgery. As the result, objective determination of the lesion size in the histopathology was impossible. Historical evidence indicates that ILC is so rare and there is little archived data that describes it fully. Studies available for review are usually limited by any combination of these three elements: study limited to only one modality, group of study participants is small, or the histological type of BC is not taken into consideration (17, 47).

## Conclusions

ILC poses a significant challenge to cancer diagnostics and management due to its histopathological and imaging complexity. This study strengthens the existing body of evidence, indicating that it is currently not possible to predict ILC molecular type when using imaging alone. Nevertheless, the large number of patients in this study made it possible to identify some radiological features that correlate to histopathology and part of the molecular panel. This study found MRI is still the preferred method for diagnosing ILC for multiple reasons that include how it enables detection of multifocal and bilateral neoplasms and allows for more reliable assessment of lesion size, both of which allow for improvements to therapeutic management plans based on TNM classification for ILC. Results obtained in the study group show, however, that there is no association between the studied parameters and proof that the morphology of ILC in imaging is independent of the cancer’s histological type if luminal A and luminal B subtypes are considered. This study, like the study of Zhiqi Yang and Xiaofeng Chen (with others authors), suggests the importance of future study on larger groups of patients in multicenter settings as well as the value of developing radiogenetics, especially due to different results of studies (48).

## Data availability statement

The original contributions presented in the study are included in the article/supplementary material. Further inquiries can be directed to the corresponding author/s.

## Ethics statement

The study was conducted according to the Declaration of Helsinki. In each case an informed consent was obtained for all the procedures. All patients provided a written informed consent for the collection and publication of their medical data. Since the study was a retrospective analysis and did not involve any experimental interventions an independent ethics committee approval was abandoned. The Institutional Review Board reviewed and approved the study.

## Author contributions

BD-K, ML, BS, and PK contributed to conception and design of the study. BD-K organized the database. MK performed the statistical analysis. BD-K and ML wrote the first draft of the manuscript. HM-J, MC, and MB wrote sections of the manuscript. All authors contributed to manuscript revision, read, and approved the submitted version.

## Funding

This research was financed through statutory subsidies by the Minister of Science and Higher Education as part of the research grant SUBZ.C280.22.001 (record number in the Simple System).

## Conflict of interest

The authors declare that the research was conducted in the absence of any commercial or financial relationships that could be construed as a potential conflict of interest.

## Publisher’s note

All claims expressed in this article are solely those of the authors and do not necessarily represent those of their affiliated organizations, or those of the publisher, the editors and the reviewers. Any product that may be evaluated in this article, or claim that may be made by its manufacturer, is not guaranteed or endorsed by the publisher.

## Abbreviations

BPE, background parenchymal enhancement; ER, estrogen receptor; FGT, fibroglandular tissue; HER2, Herceptin receptor; IDC, invasive ductal carcinoma; ILC, invasive lobular carcinoma; Lum A, luminal A; Lum B, luminal B; MMG, mammography; NME, non-mass enhancement; PGR, progesterone receptor; TN, triple negative.
